# Soluble amyloid beta-containing aggregates are present throughout the brain at early stages of Alzheimer’s disease

**DOI:** 10.1093/braincomms/fcab147

**Published:** 2021-07-02

**Authors:** Dimitrios I Sideris, John S H Danial, Derya Emin, Francesco S Ruggeri, Zengjie Xia, Yu P Zhang, Evgeniia Lobanova, Helen Dakin, Suman De, Alyssa Miller, Jason C Sang, Tuomas P J Knowles, Michele Vendruscolo, Graham Fraser, Damian Crowther, David Klenerman

**Affiliations:** 1 Yusuf Hamied Department of Chemistry, University of Cambridge, Cambridge CB2 1EW, UK; 2 Neuroscience, Research and Early Development, Biopharmaceuticals R&D, AstraZeneca, Cambridge CB21 6GH, UK; 3 Laboratories of Organic and Physical Chemistry, Wageningen University, Wageningen 6703 WE, Netherlands; 4 Cavendish Laboratory, University of Cambridge, Cambridge CB3 0H3, UK; 5 UK Dementia Research Institute at Cambridge, Cambridge CB2 0XY, UK

**Keywords:** Alzheimer’s disease, neurodegeneration, neuroinflammation, amyloid beta 42, soluble aggregates

## Abstract

Protein aggregation likely plays a key role in the initiation and spreading of Alzheimer’s disease pathology through the brain. Soluble aggregates of amyloid beta are believed to play a key role in this process. However, the aggregates present in humans are still poorly characterized due to a lack of suitable methods required for characterizing the low concentration of heterogeneous aggregates present. We have used a variety of biophysical methods to characterize the aggregates present in human Alzheimer’s disease brains at Braak stage III. We find soluble amyloid beta-containing aggregates in all regions of the brain up to 200 nm in length, capable of causing an inflammatory response. Rather than aggregates spreading through the brain as disease progresses, it appears that aggregation occurs all over the brain and that different brain regions are at earlier or later stages of the same process, with the later stages causing increased inflammation.

## Teaser

Sideris et al. report that soluble inflammatory amyloid beta-containing aggregates are found in all regions of early Alzheimer’s disease brain. Aggregation may therefore be occurring all over the brain simultaneously, with different brain regions being in different stages of the same process, with the later stages causing increased inflammation.

## Graphical Abstract

**Graphical Abstract:**
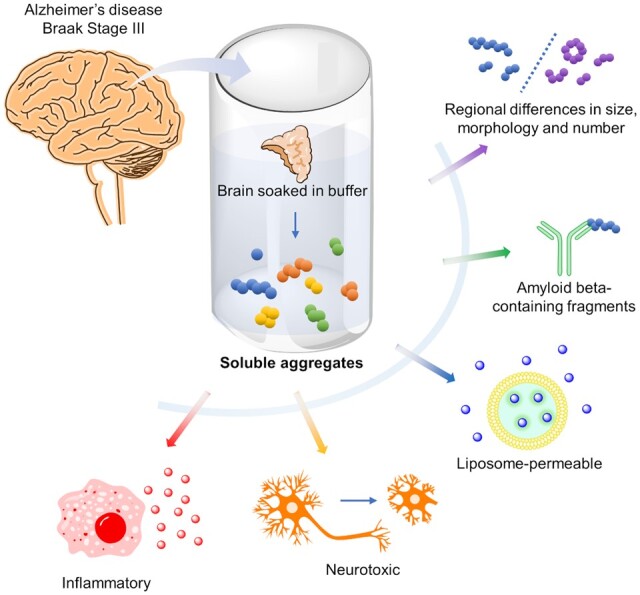

## Introduction

Alzheimer’s disease is a progressive neurodegenerative disease characterized by memory loss and cognitive decline. It is the leading cause of dementia, which is currently the leading cause of death in the UK.^1^ The aggregation of the amyloid beta 42 peptide (Aβ42) is believed to play a key role in the initiation and development of the disease.^2^ It is widely accepted that small soluble Aβ42 aggregates are toxic, likely through a variety of mechanisms, including the permeabilization of cell membranes through non-specific binding and by specific binding to pattern-recognizing membrane receptors.^3–6^ This can lead to microglial activation and inflammation,^7^ a key player in Alzheimer’s disease pathology.^6^^,^^8^^,^^9^ Microglial activation increases as the disease progresses, and they become dystrophic at late stages.^10^ Activated microglia have been shown to induce astrocytes into releasing neurotoxic factors.^11^ Through these toxic mechanisms, Aβ42 aggregates have been shown to cause neuronal cell death, synaptic dysfunction, as well as cognitive impairment in Alzheimer’s disease patients and animal models of the disease.^12–22^

Despite only being a small subset of the overall protein aggregates found in brain tissue, it is believed that the soluble aggregates are responsible for most of the toxicity.^23^ This is supported by the fact that insoluble amyloid plaque counts do not correlate well with cognitive function,^24–27^ and soluble Aβ levels correlate better with cognition than insoluble Aβ levels.^28–32^ Soluble aggregates have been found in the brain lysates of Alzheimer’s disease patients.^33–35^ However, these soluble aggregates occur at low concentrations and hence have been poorly characterized due to a lack of sensitive methods, with many more studies performed on aggregates formed from synthetic Aβ42, since they are available in higher concentrations.^36^ It is still unclear how comparable endogenous aggregates from Alzheimer’s disease patients are to synthetic aggregates or to those from animal models.^37^^,^^38^ Far less research has been done on the aggregates in human CSF or extracted from post-mortem brain. Brain samples are generally homogenized and hence include large amounts of insoluble aggregates, which are largely inert, as well as soluble aggregates potentially complicating the interpretation of any analysis.

To address these issues, there has been a recent effort to selectively extract the soluble aggregates from human brain tissue using minimally perturbative methods.^23^^,^^39^ We have developed a suite of sensitive methods with the potential to characterize aggregates and measure their properties.^7^ These include correlating changes in the aggregate size distribution with changes in the mechanism of toxicity as demonstrated by experiments on CSF from Alzheimer’s disease patients.^40^ In the literature, soluble aggregates from Braak stage VI AD brain have been found to contain Aβ42 aggregates as identified through western blotting and ELISAs. These samples have also been shown to cause neurite length retraction on iPSC-derived neurons, and can block synaptic long-term potentiation.^23^^,^^41^ Furthermore, they induce neuronal hyperactivation in mouse CA1 hippocampal neurons, as seen with two photon Ca^2+^ imaging.^42^^,^^43^ Toxicity caused by soluble aggregates is believed to be mediated partly by prion protein.^44^ Experiments have shown that soluble aggregates extracted from soaking brain tissue are as toxic as homogenized brain samples but contain significantly less Aβ42 aggregates making them an ideal sample for characterization. This toxicity appears to be Aβ-dependent, as evidenced by Aβ-immunodepleted samples being significantly less toxic.^23^ However, there is no information about the size or structure of these aggregates extracted by soaking post-mortem brain nor how the aggregates differ between different brain regions.

Alzheimer’s disease has a typical pathological progression, starting in the hippocampal/entorhinal cortex regions and spreading to the temporal, parietal and frontal lobes before affecting the occipital lobe.^45^^,^^46^ For our initial experiments, we decided to study soluble aggregates from Braak stage III, which is at the early stages of pathological progression and therefore before the appearance of global pathology, to assess regional variability between different regions of the same patient and between patients.^47^^,^^48^ After establishing that our assays have sufficient sensitivity to detect the aggregates present, we characterized the soluble aggregates from eight brain regions, from three Alzheimer’s disease patients. We then chose to compare in more detail the soluble aggregates from two distinct regions, the hippocampus (HPC), which is significantly affected early in the disease, and the visual association cortex (VAC), a region affected later in the disease, with the latter acting as an internal control for each brain.

**Figure 1:**
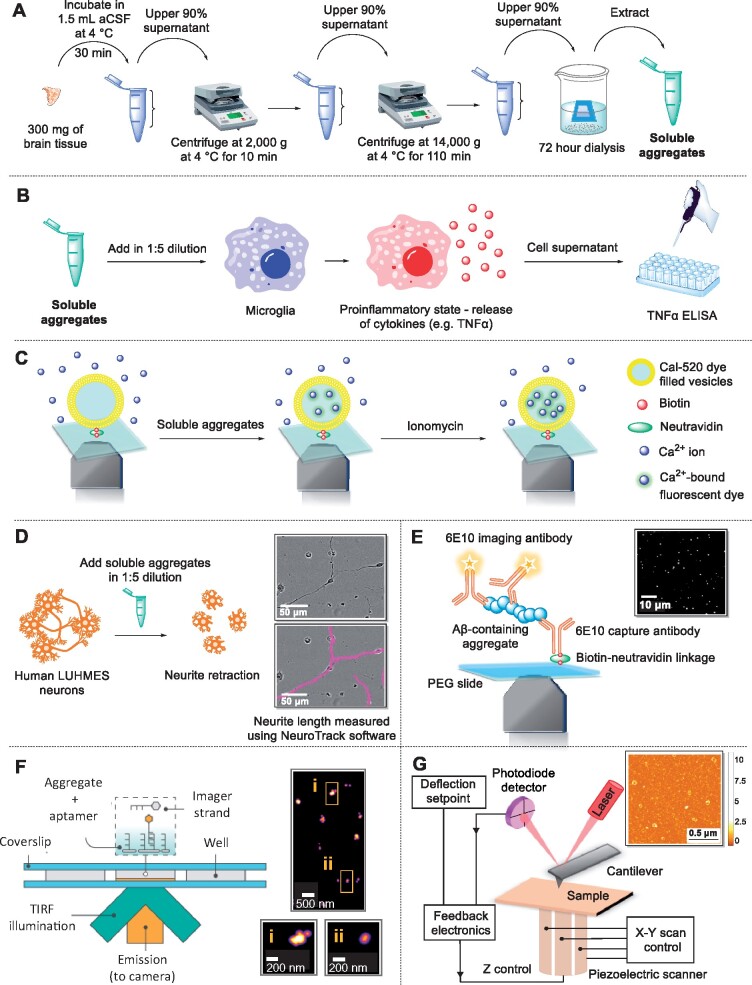
**Schematics of assays used to characterize soluble aggregates.** (**A**) Extraction of soluble aggregates from human brain tissue through soaking in aCSF. (**B**) Neuroinflammation assay using BV2 cells to measure production of TNF-α. (**C**) Liposome assay used to measure the aggregates’ ability to penetrate a lipid membrane. (**D**) Neurite length assay used to measure neurotoxicity. (**E**) Single-molecule pull-down imaging used for the characterization of Aβ-containing aggregates. (**F**) Aptamer DNA-PAINT imaging used to characterize the size and number of aggregates. (**G**) AFM imaging of 3D morphology used to characterize morphology and cross-sectional dimensions of the aggregates. aCSF, artificial cerebrospinal fluid; TNF-α, tumour necrosis factor-α, ELISA, enzyme-linked immunosorbent assay; TIRF, total internal reflection fluorescence.

In this pilot study, we applied our methods (Fig. 1) to characterize soluble aggregates from Braak stage III (Table 1). We have identified the similarities and differences between the soluble aggregates in eight different regions by providing a detailed characterization of their size, morphology, structure, neurotoxicity, inflammatory potential and capability to permeabilize a lipid membrane. These data show that soluble aggregates of a range of sizes and morphologies, capable of causing inflammation, are already present in all brain regions at Braak stage III and that aggregation is occurring by the same processes all over the brain to a greater or lesser extent.

**Table 1:** Patient information

Patient	ID No.	Age at PM (years)	PMI (h)	Gender (M/F)	Cause of death	Braak stage
AD1	NP17-216	77	55	F	Burkitt's Lymphoma	III
AD2	NP17-194	71	70	M	Pneumonia	III
AD3	NP17-020	88	44	M	End stage dementia	III

AD, Alzheimer’s disease; F, Female; M, Male; PM, post-mortem; PMI, post-mortem interval.

Information from three Braak stage III Alzheimer’s disease patients (AD1, AD2 and AD3) whose brain tissue has been analysed in this study.

## Materials and methods

### Alzheimer’s disease brain tissue

Fresh frozen brains from three Alzheimer’s disease patients (Table 1) were received whole from the Addenbrooke’s post-mortem room, or from other centres around the country. Transport and consent details were reviewed and handled by the Cambridge Brain Bank. Processing of tissue was carried out in Addenbrooke’s hospital, where regions of interest were removed from the left cerebral hemisphere and frozen at −80°C. The brains used for the following experiments were diagnosed as being at Braak stage III by histopathologists, based on tau protein pathology.

### Extraction of soluble aggregates from human brain tissue

Soluble aggregates were obtained by following a previously established protocol with a few adaptations.^23^ Briefly, human brain tissue was chopped into 300 mg pieces using a razor blade and incubated with gentle agitation in 1.5 ml of artificial cerebrospinal fluid (aCSF) buffer (124 mM NaCl, 2.8 mM KCl, 1.25 mM NaH_2_PO_4_, 26 mM NaHCO_3_; pH 7.4, supplemented with 5 mM EDTA, 1 mM EGTA, 5 *μ*g/ml leupeptin, 5 *μ*g/ml aprotinin, 2 *μ*g/ml pepstatin, 20 *μ*g/ml Pefabloc, 5 mM NaF) at 4°C for 30 min. Samples were centrifuged at 2000 *g* at 4°C for 10 min and the upper 90% of the supernatant was collected and centrifuged at 14 000 *g* for 110 min at 4°C. The upper 90% of the supernatant was extracted and dialyzed using Slide-A-Lyzer™ cassettes (Thermo Scientific, Cat. 66330) with a 2 kDa molecular weight cut off, against 100-fold excess of fresh aCSF buffer with gentle agitation at 4°C. Buffer was changed three times over the course of 72 h dialysis. The prep was carried out under sterile conditions, using autoclaved LoBind Eppendorf tubes and pre-sterilized pipette tips to reduce endotoxin contamination. Samples were aliquoted into small volumes, snap frozen and stored in a −80°C freezer and thawed only once prior to experimentation.

### Neuroinflammation assay

BV-2 cells derived from immortalized murine neonatal microglia (European Collection of Authenticated Cell Cultures) were grown in T25 flasks in Dulbecco’s modified eagle medium (DMEM) (Gibco, Life Technologies, Cat. 21063-029) with 10% (v/v) foetal bovine serum (FBS) (Sigma-Aldrich, St. Louis, MO, Cat. F0926), 100 U/ml penicillin/100 *μ*g/ml streptomycin (Gibco, Life Technologies, Cat. 15140-122), 2 mM l-glutamine (Gibco, Life Technologies, Cat. 25030-024), 1% (v/v) sodium pyruvate and 1% (v/v) HEPES buffer. They were grown in a humidified environment, incubated at 37°C, with 5% CO_2_, 95% air. Cells were plated in flat-bottom 96-well plates (Corning, Costar, Cat. CLS3997) in DMEM 10% (v/v) FBS at a concentration of 1.65 × 10^5^ cells/ml (150 *μ*l per well). Twenty-four hours after plating, the cells were washed with fresh pre-warmed (37°C) media and kept in phenol red-containing DMEM 1% (v/v) FBS. Cells were treated with soluble aggregates in a 1:5 dilution. Lipopolysaccharide (Invivogen San Diego, CA, Cat. Tlrl-3pelps) at 10 ng/ml was used as a positive control and aCSF buffer in a 1:5 dilution was used as a negative control. The supernatant was collected every 24 h for analysis and the wells were washed with fresh media and replaced with fresh solution. The supernatant was stored in a −80°C freezer and thawed only once before being measured with a mouse TNF-α DuoSet ELISA (R&D Systems, MN, USA, Cat. DY410) using a plate reader (CLARIOstar, BMG Labtech, Ortenberg, Germany) at 450 nm. Experiments were carried out over 96–120 h. Three wells were used for each sample of soluble aggregates to estimate variation.

### Cytotoxicity assay

Cell supernatant collected from the neuroinflammatory assay was stored at −80°C. A lactate dehydrogenase (LDH) assay (Abcam, Colorimetric, Cat. ab102526) was used to detect the concentration of LDH (mU/ml) in the supernatants. Cells treated with RIPA lysis buffer (Thermo Scientific, Cat. 89900) were taken as complete cell death (100%), whereas cells of the same density treated with aCSF buffer were taken as healthy cells (0%). The supernatants were thawed only once before taking measurements.

### Immunoprecipitation experiments

Immunoprecipitation was carried out as described previously, with a few modifications.^49^ Briefly, Dynabeads^®^ Protein A (Invitrogen, Cat. 10002D) and Dynabeads^®^ Protein G (Invitrogen, Cat. 10004D), mixed in a 1:1 ratio in low-binding snap top tubes (Eppendorf AG, Hamburg, Germany), were used to bind and pull down an APP-binding antibody (6E10, Mouse IgG1, Biolegend, Cat. SIG-39320). The antibody was added at a concentration of 20 *μ*g/ml. Four hundred microlitres of soluble aggregates from the HPC and VAC regions were then added to the mix. The snap top tubes were placed on a magnetic rack to pull down the magnetic beads, along with the antibody and binding targets. The neuroinflammation assay was then carried out on samples of soluble aggregates with or without immunodepletion of Aβ-containing fragments.

### Neurite length assay

Lund Human Mesencephalic (LUHMES) cells were purchased from the American Type Culture Collection (ATCC) (Cat. CRL-2927) and were cultured according to the ATCC guidelines. Briefly, the cells were grown in a T75 flask pre-coated with 50 *μ*g/ml poly-l-ornithine (Sigma, Cat. P3655) and 1 *μ*g/ml Human Fibronectin (Sigma, Cat. F-0895) in DMEM: F12 (Invitrogen, Cat. 31330038), supplemented with l-glutamine, N2 supplement (Invitrogen, Cat. 17502-048), and basic recombinant human Fibroblast Growth Factor (b-FGF) (Sigma, Cat. F0291). Experiments were carried out after 4 days of differentiation in DMEM: F12 medium containing N2 supplement, 2 ng/ml human recombinant GDNF (R&D Systems, Cat. 212-GD), 1 mM dibutyryl cAMP (Sigma, Cat. D0260) and 1 *μ*g/ml tetracycline (Sigma, Cat. 87128). Cells were plated at a density of 1 × 10^5^ cells/ml (100 *μ*l per well) and treated with soluble aggregates in a 1:5 dilution. Lipopolysaccharide (Invivogen San Diego, CA, Cat. Tlrl-3pelps) at 10 ng/ml was used as a positive control and aCSF buffer in a 1:5 dilution was used as a vehicle control. The plate was placed in an IncuCyte^®^ S3 live cell imaging system right after treatment and monitored for 48 h. Four images were taken per well (∼600 cells per field of view), every hour, with three wells per condition, totalling ∼7200 cells imaged per condition per experiment. Two biological replicates were carried out. Neurite length was measured using NeuroTrack software with the following settings: Segmentation Mode: Brightness; Segmentation Adjustment: 0.7; Adjust size (pixels): 1; Min Cell Width (*μ*m): 25; Area (*μ*m^2^) min: 500; Neurite Filtering: Best; Neurite Sensitivity: 0.25; Neurite Width (*μ*m): 4.

### Membrane permeabilization assay

The membrane permeabilization assay was performed as described previously.^50^ Briefly, liposomes composed of 16:0–18:1 PC and 18:1–12:0 biotin PC (100:1) (Avanti Lipids), with an average diameter of 200 nm, were prepared using extrusion and freeze-thaw cycles. Vesicles filled with 100 *μ*M Cal-520 dye were bound to a glass surface coated with PLL-g-PEG and PLL-g-PEG biotin (10:1) (Susos AG), via a biotin-neutravidin linkage. A series of 9 different images were taken of 30 *μ*l Ca^2+^ containing buffer (L-15) alone to measure the background for each set (*F*_blank_). The same volume of soluble aggregates (50 *μ*l) was then incubated on the glass coverslip for 15 min and imaged in the exact same fields of view (*F*_sample_). The same fields of view were then re-imaged after the addition of 10 *μ*l of 50 *μ*g/ml ionomycin (*F*_ionomycin_). By first determining the intensity of each individual vesicle, the average Ca^2+^ influx was calculated using the following formula:

$$\left(\frac{F_{\mathrm{sample}}–F_{\mathrm{blank}}}{F_{\mathrm{ionomycin}}-F_{\mathrm{blank}}}\right)\times 100\%$$

Imaging was carried out using a home-built total internal reflection fluorescence (TIRF) microscope, fitted with a 488 nm laser (Toptica, iBeam smart, 200 mW, Munich, Germany), which was used to excite the samples. The laser beam was expanded and collimated using two Plano-convex lenses on the back-focal plane of the 60×, 1.49 NA oil immersion objective lens (APON60XO TIRF, Olympus, product number N2709400) to a spot of adjustable diameter. An EmCCD camera (Photometrics Evolve, EVO-512-M-FW- 16-AC-110) was used to image the dye fluorescence emissions collected by the objective.

### Aptamer DNA-PAINT imaging

Aptamer DNA-PAINT imaging was performed as described previously, with a few adaptations.^51^ Briefly, round slides were cleaned for 1 h with argon plasma. A multiwell chamber coverslip (CultureWell CWCS-50R-1.0) was then added to the slide. The wells were cleaned with PBS 1% (v/v) Tween 20 for 1 h before adding 5× diluted soluble aggregates in PBS for 1 h. The wells were washed twice with fresh PBS and replaced with imaging mix [2 nM imaging strand (sequence CCAGATGTA-TCY3B), and 100 nM aptamer-docking strand (sequence GCCTGTGGTGTTGGG-GCGGGTGCGTTATACATCTA) in PBS]. All buffers were passed through a 0.02 *μ*m filter (Anotop25, Whatman, Cat. 516-1501) before use. Prior to imaging, a clean coverslip was used to seal the wells to prevent evaporation. Imaging was performed on a home built TIRF microscope using a 1.49 N.A., 60× objective (UPLSAPO, 60X, TIRF, Olympus) and a perfect focus system. More details about the microscope set up and data analysis are described in Whiten et al.^51^

### Single-molecule pull-down imaging

Glass coverslips covalently mounted with polyethylene glycol (PEG) were used for single-molecule pull-down (SiMPull) experiments. Coverslip preparation was carried out as described previously, with a few modifications.^52^ Briefly, glass coverslips (26 × 76 mm, thickness 0.15 mm, Thermo Scientific) were washed ultrasonically (cleaner USC100T, VWR), in a series of solvents [10 min in 18.2-MΩ cm^−^^1^ Milli-Q water, 10 min in acetone, then 10 min in methanol (MeOH)]. The washed coverslips were then etched by 1 M potassium hydroxide (KOH) under 20 min ultrasonication and rinsed with a series of solvents (MeOH, 18.2-MΩ cm^−1^ Milli-Q water, then MeOH). The processed coverslips were dried using nitrogen flow and cleaned with argon plasma for 15 min (Femto Plasma Cleaner; Diener Electronic). The coverslips were then silanized with 5 ml of 3-aminopropyl triethoxysilane (Fisher Scientific UK, Cat. 10677502), 8.3 ml acetic acid (AcOH) in 166.7 ml MeOH for 20 min, with 1 min ultrasonication at the start and mid-point of reaction (10 min after the start point). The silanized coverslips were then rinsed in MeOH, 18.2-MΩ cm^−1^ Milli-Q water, followed by MeOH and dried using nitrogen flow. 50-well polydimethylsiloxane (PDMS) gaskets (Sigma, GBL103250-10EA) were then attached to the cleaned and silanized coverslips. To passivate the wells, 9 µl of a 100:1 aqueous mixture of succinimidyl valeric acid PEG (MPEG-SVA-5000) (110 mg ml^−1^, Laysan Bio Inc.) and Biotin-PEG-SVA-5000 (1.1 mg ml^−1^, Laysan Bio Inc.) were added, with additional 1 µl of 1 M sodium bicarbonate (NaHCO_3_) (pH 8.5). The coverslips were incubated with PEG solution overnight in a humid chamber and then rinsed with 18.2-MΩ cm^−1^ Milli-Q water and dried with nitrogen flow. The passivated wells were treated by adding 9 µl of MS(PEG)4 methyl-PEG-NHS-Ester (10 mg ml^−1^, Thermo Scientific, Cat. 22341), with additional 1 µl of 1 M NaHCO_3_ (pH 8.5). The coverslips were incubated with PEG solution overnight in a humid chamber and then rinsed with 18.2-MΩ cm^−1^ Milli-Q water and dried with nitrogen flow. PEGylated glass coverslips were stored in a desiccator at −20°C until needed.

For the experiment, neutravidin (0.2 mg/ml) was added to the coverslip for 5 min, followed by two wash steps with 0.05% (v/v) PBST and once with 1% (v/v) PBST. Afterwards biotinylated 6E10 (Signet, Cat. 9340-02, 10 nM) was added for 10 min, followed by two wash steps with 0.05% (v/v) PBST and once with 1% (v/v) PBST. The soluble aggregates were added for at least 1 h at room temperature followed by two wash steps with 0.05% (v/v) PBST and once with 1% (v/v) PBST. The coverslips were blocked using blocking solution containing 0.1% (w/v) bovine serum albumin (BSA) (Thermo Scientific, Cat. AM2616), 10% (v/v) salmon sperm (Thermo Scientific, Cat. 15632011) and 0.1% (v/v) PBST for 1 h at room temperature. The coverslips were then incubated with labelled 6E10 (cat. 80302, 500 pM) for 45 min, followed by three washing steps with 0.05% (v/v) PBST. To properly seal the imaging chamber and prevent evaporation, 3 *μ*l of PBS was added to each well and sandwiched with a second coverslip.

To determine the number of fluorescent molecules in each image, a *z*-stack was generated in ImageJ. The images were cropped to 380 × 380 pixels and the contrast was adjusted. Using the negative control as a baseline, a threshold was applied to all images, and single molecules above this threshold were counted.

### Atomic force microscopy

Samples of soluble aggregates were diluted 10× in PBS buffer and imaged on freshly cleaved mica substrates using atomic force microscopy (AFM). Ten microlitres diluted samples were deposited on the substrate at room temperature. The samples were incubated for 10 min, followed by rinsing with 1 ml milliQ water. The samples were then dried using a gentle flow of nitrogen gas. AFM maps of 3D morphology of all the samples were acquired in regime of constant phase change, with 2–4 nm/pixel resolution using a NX10 (Park Systems, city, South Korea) operating in non-contact mode.^53^ This set up was equipped with a silicon tip with a nominal radius of <10 nm and spring constant of 5 N/m (PPP-NCHR). For each sample, we scanned an area between 250 and 500 *μ*m^2^. The lower limit was used for samples where aggregated species were found and the upper limit for the samples without aggregates. Scanning Probe Image Processor (SPIP) (version 6.7.3, Image Metrology, Denmark) software was used for image flattening and single aggregate statistical analysis. The average level of noise for each image was measured using SPIP software and was smaller than 0.1 nm.^54^ All the measurements were performed at room temperature.

### Statistical analysis

GraphPad Prism v9 was used to carry out statistical analyses for all experimental data except for AFM data. Unpaired two-tailed *t*-tests have been used to test the null hypothesis, in cases where two independent, normally distributed samples needed comparing (liposome assay, immunoprecipitations, and Aptamer-DNA PAINT data). An alpha value of (*P* < 0.05) was chosen to represent significant differences in the data (* = *P* ≤ 0.05, ** = *P* ≤ 0.01, *** = *P* ≤ 0.001). In cases where more than two independent variables needed comparing, a one-way analysis of variance (ANOVA) was used (neuroinflammation, neurite length assay). This was followed by a Tukey *post hoc* test when comparing the mean of each variable to the mean of every other variable, or a Dunnett *post hoc* test when comparing the mean of each variable to the mean of one control variable. Kolmogorov–Smirnov tests have been used when comparing non-normally distributed cumulative distributions (Aptamer-DNA PAINT and SiMPull data). Individual statistical values are reported in the figure legends. To assess the variability between independent repeats of the experiment and between patients, multiple comparisons tests were carried out (Supplementary Tables 1 and 2).

Scanning Probe Image Processor (SPIP) software was used for image flattening and single aggregate statistical analysis for AFM imaging data. AFM data were plotted using OriginPro^®^ 2021. Mann–Whitney two-tailed tests were used to compare the medians of the non-normally distributed data sets. Individual statistical values are reported in the figure legends.

Patient samples were blinded prior to experimentation and were only unblinded after data analysis. Region selection for all imaging experiments was automated, ensuring randomization and elimination of human bias. Furthermore, data analysis parameters (e.g. thresholding) were kept consistent within each data set.

Data gathered by ELISA were analysed using MARS data analysis software. A four-parameter logistic fit was fitted to the data, as per the kit manufacturer’s instructions.

### Data availability

Raw data were generated at the Yusuf Hamied Department of Chemistry, University of Cambridge. Derived data supporting the findings of this study are available from the corresponding author on request.

## Results

### Characterization of soluble aggregates using a series of sensitive assays

A series of sensitive assays were employed to characterize brain-derived soluble aggregates by measuring their neuroinflammatory potential, liposome-permeability, neurotoxicity, size, number and morphology (Fig. 1).

### Global inflammation in the brain of Braak stage III patients

**Figure 2:**
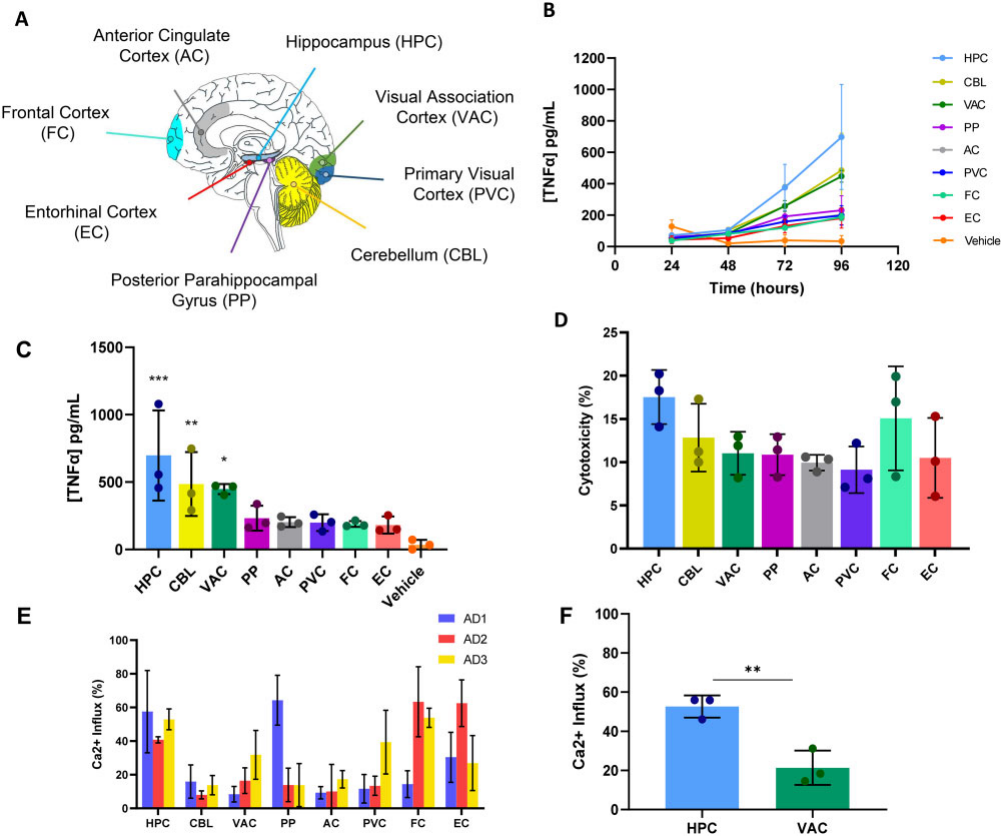
**Inflammation and liposome data.** (**A**) Diagram of the eight regions that were removed, soaked, and used for experimentation. (**B**) TNF-α response from BV2 cells treated with soluble aggregates (diluted 1:5) from eight different brain regions. Each point represents an average from three different Alzheimer’s disease patients. Vehicle control was aCSF at equal volume to soluble aggregates. Lipopolysaccharide at 10 ng/ml was used as a positive control (not shown). Connecting lines have been added for visual clarity. Error bars are mean ± SD. Individual patient data can be found in Supplementary Fig. 1. (**C**) TNF-α measurements from the 96-h time point of the inflammation assay. Each point represents one of the three Alzheimer’s disease patients. Error bars are mean ± SD. Statistical analysis has been carried out relative to the Vehicle control [one-way ANOVA: *F*(8,18) = 5.989, *P* < 0.001. *Post hoc* Dunnett test against Vehicle: HPC: *P* < 0.001; CBL: *P* = 0.008; VAC: *P* = 0.02; PP: *P* = 0.46; AC: *P* = 0.62; PVC: *P* = 0.64; FC: *P* = 0.69; EC: *P* = 0.74]. (**D**) Cell viability was assessed from cell supernatant from the 96-h time point of the inflammation assay using an LDH assay. Each point represents one of three Alzheimer’s disease patients. Error bars are mean ± SD. (**E**) Ca^2+^ influx of each patient and region measured by liposome assay. Error bars are mean ± SD of fields of view. HPC and VAC samples have been repeated in an independent experiment and show a similar trend. (**F**) Ca^2+^ influx from two repeat experiments, averaged by brain region (HPC and VAC). Each point represents one of three Alzheimer’s patients. Error bars are mean ± SD (HPC vs VAC: *n* = 3, *P* = 0.007, *t*_4_ = 5.201). AC, anterior cingulate cortex; CBL, cerebellum; EC, entorhinal cortex; FC, frontal cortex; HPC, hippocampus; PP, posterior parahippocampal gyrus, PVC, primary visual cortex; TNF-α, tumour necrosis factor-α; VAC, visual association cortex.

Soluble aggregates were extracted from eight different brain regions from three Alzheimer’s disease patients (Fig. 2A). Samples from all regions appear to be neuroinflammatory (Fig. 2B and C), cytotoxic (Fig. 2D) and capable of permeabilising liposomes (Fig. 2E), to varying degrees. This suggests that there is global pathology even at Braak stage III. Despite patient-to-patient variability (Supplementary Fig. 1), hippocampal aggregates appear to be the most toxic in all three patients and have therefore been further characterized with our assays in comparison to the VAC, a region that is generally affected later in Alzheimer’s disease progression. HPC aggregates caused a significantly higher Ca^2+^ influx than VAC aggregates (Fig. 2F), suggesting they may be better at permeabilizing liposomes.

### Aβ-containing fragments are likely driving the soluble aggregate inflammatory response

**Figure 3:**
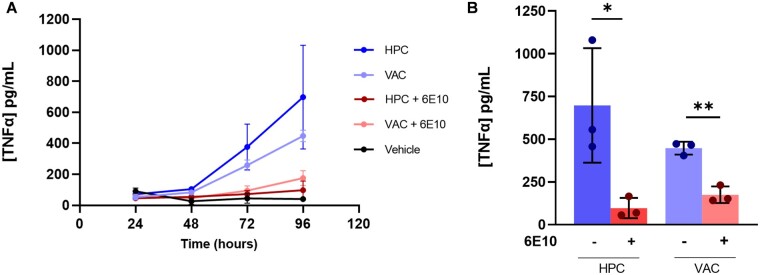
**Immunodepletion of Aβ-containing fragments.** (**A**) TNF-α response measured from BV2 cells treated with soluble aggregates that have either undergone (red) or not undergone (blue) a pull-down using a 6E10 APP-binding antibody. Each point represents an average from three patients. Vehicle control was aCSF at equal volume to soluble aggregate samples. Lipopolysaccharide at 10 ng/ml was used as positive control (not shown). Error bars are mean ± SD. (**B**) TNF-α measured at the 96-h time point. Each point represents one of three Alzheimer’s disease patients. Error bars are mean ± SD (Unpaired two-tailed *t*-test, HPC vs HPC + 6E10: *n* = 3, *P* = 0.04, *t*_4_ = 3.061; VAC vs VAC + 6E10: *n* = 3, *P* = 0.002, *t*_4_ =7.658). Individual patient data can be found in Supplementary Fig. 2.

The solutions extracted from brain tissue are heterogeneous mixtures of proteins. In order to identify whether Aβ-containing fragments were involved in the inflammatory response (Fig. 2B and C), an immunoprecipitation with an APP-binding antibody (6E10) was carried out. This enabled the treatment of BV2 cells with samples of soluble aggregates with and without immunodepletion of Aβ-containing fragments. Samples immunodepleted of Aβ-containing fragments using the 6E10 antibody caused a significantly lower inflammatory response than samples that did not undergo immunodepletion (Fig. 3A, B and Supplementary Fig. 2). These data are consistent with Aβ aggregates being involved in neuroinflammation.

### Soluble aggregates cause neurite retraction in human LUHMES neurons

**Figure 4:**
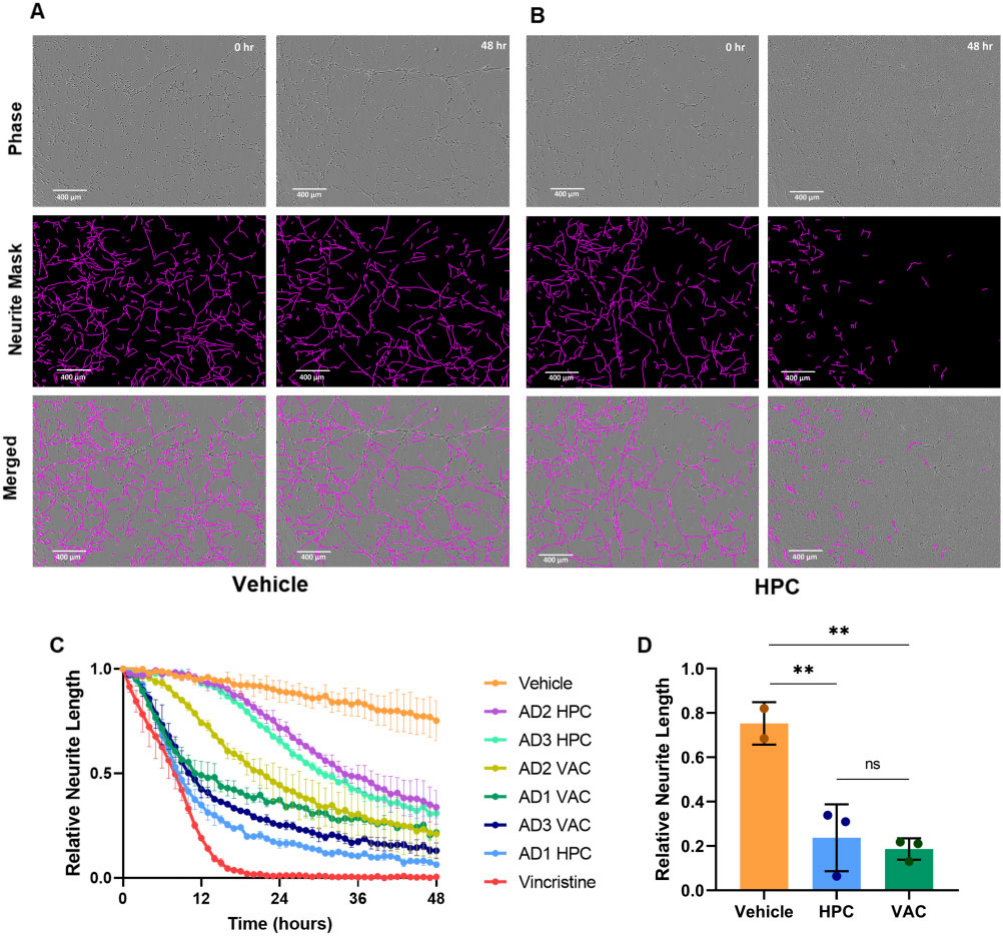
**Neurite length.** Representative images of neurite length of LUHMES cells treated with(**A**) aCSF buffer and (**B**) HPC at 0 and 48-h time points. Representative images of LUHMES cells treated with HPC and VAC from all three Alzheimer’s disease patients can be found in Supplementary Fig. 3. (**C**) Relative neurite length of LUHMES cells (∼7200 cells imaged per condition) treated with soluble aggregates at a 1:5 dilution for 48 h, normalized to neurite length at the 0-h time point (1.0), and vincristine at 48 h (0) to signify total neurite retraction. Vehicle was aCSF at same dilution as soluble aggregates (1:5), and vincristine (50 nM) served as a positive control for neurite retraction. Error bars are mean ± SD from two biological repeats, with each condition carried out in triplicate wells, and 4 images analysed per well. (**D**) Relative neurite length at the 48-h time point averaged by brain region. Each point represents one of three Alzheimer’s disease patients. Error bars are mean ± SD [One-way ANOVA: *F*(2,5) = 18.63, *P* = 0.005. *Post hoc* Tukey test: *n* = 3 for all: Vehicle vs HPC: *P* = 0.008; Vehicle vs VAC: *P* = 0.005; HPC vs VAC: *P* = 0.84].

Treatment of LUHMES cells with HPC and VAC soluble aggregates caused significant neurite retraction over 48 h (Fig. 4A–C and Supplementary Fig. 3). This suggests that the soluble aggregates are neurotoxic, similar to what has been previously reported with Braak stage VI soluble aggregates.^23^ Surprisingly, there was no significant difference between the level of HPC and VAC aggregate-induced neurite retraction (Fig. 4D).

### Length and number characterization of soluble aggregates

**Figure 5:**
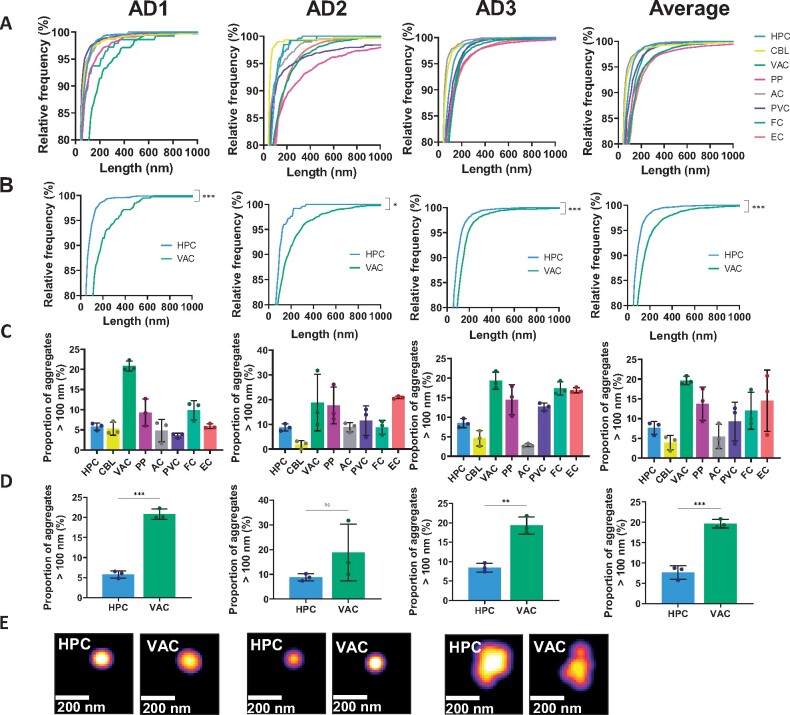
**Length and number characterization of soluble aggregates using Aptamer DNA-PAINT.** (**A**) Cumulative frequency of soluble aggregates in all eight brain regions and in (**B**) HPC and VAC regions only, from three independent experiments (Kolmogorov–Smirnov test, AD1: *P* < 0.001, D = 0.2092; AD2: *P* = 0.03, D = 0.0974; AD3: *P* < 0.001, D = 0.3799; Average: *P* < 0.001, D = 0.2016). See Supplementary Table 1 for the results of multiple comparison test. (**C**) Proportion of soluble aggregates over 100 nm in length in all eight brain regions and in (**D**) HPC and VAC regions only. Each point in the AD1, AD2 and AD3 graphs represents one of three replicates; each point in the Average graph represents one of three Alzheimer’s disease patients. Error bars are mean ± SD. (Unpaired two-tailed *t*-test, *n* = 3 for all. AD1: *P* < 0.001, *t*_4_ = 16.71; AD2: *P* = 0.21, *t*_4_ = 1.510; AD3: *P* = 0.002, *t*_4_ = 7.618; Average: *P* < 0.001, *t*_4_ = 10.66). (**E**) Representative super-resolved images of aggregates in HPC and VAC samples.

The size and number of the soluble aggregates have been characterized by Aptamer DNA-PAINT super-resolution microscopy. This was performed using an aptamer that binds to fibrillar Aβ, although it can also bind fibrillary α-synuclein fibrils. The aggregates vary from region to region, and from patient to patient. In all cases, there is a variety of soluble aggregates of a range of lengths detected, however the length distributions differ (Fig. 5A and C). The HPC aggregates appear to be shorter than VAC aggregates in general, and have a smaller proportion of longer aggregates (over 100 nm) (Fig. 5B and D). Despite the clear difference in length there is a relatively small difference between the inflammatory responses of these two regions (Fig. 2B), suggesting that the aggregates smaller than 100 nm (80–95% of all aggregates) are most inflammatory.

### Structural characterization of aggregates using AFM

**Figure 6:**
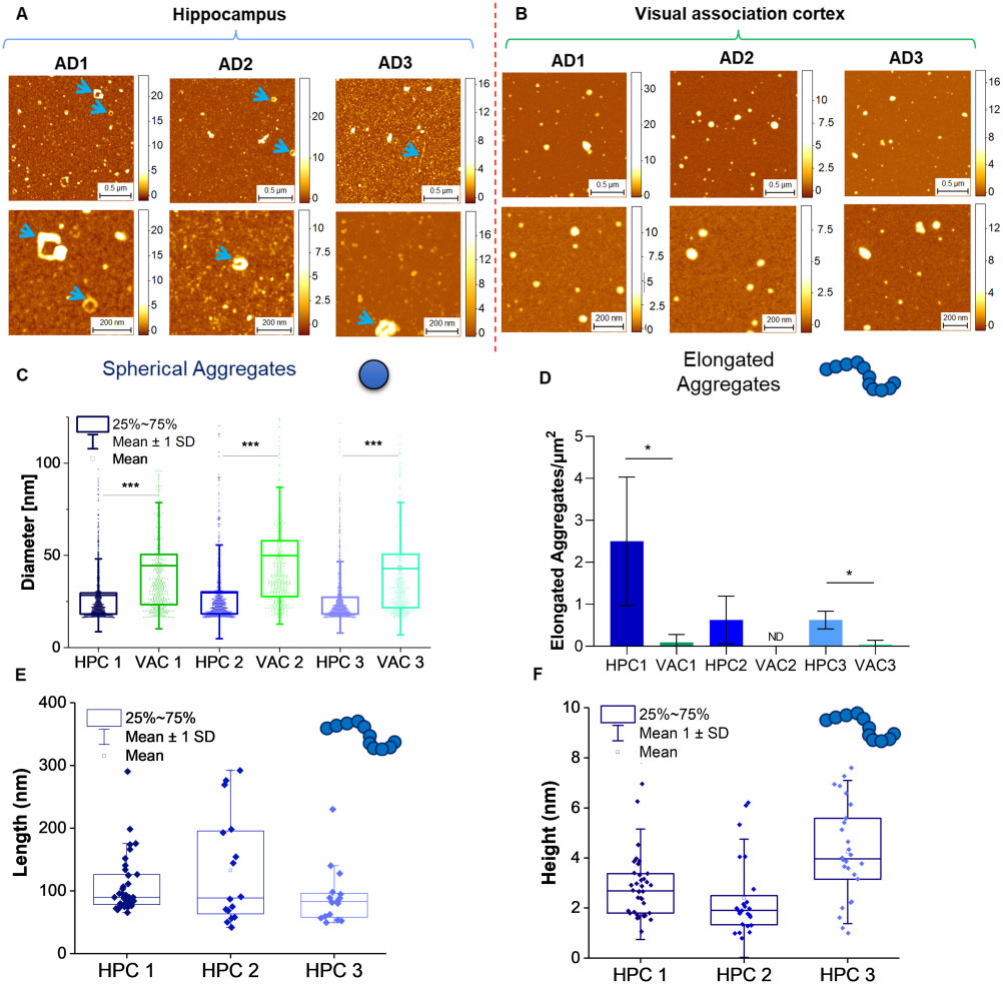
**High-resolution AFM imaging of aggregates 3D morphology.** Structural characterization of the aggregates in (**A**) HPC and (**B**) VAC regions using AFM. The blue arrows are highlighting toroidal and elongated aggregates. (**C**) The single aggregate statistical analysis of the cross-sectional diameter of the spherical aggregates reveals that VAC spherical aggregates are significantly larger than HPC ones (Mann–Whitney test, two-tailed; HPC1 vs VAC1: *P* < 0.001, U = 140761; HPC2 vs VAC2: *P* < 0.001, U = 56141; HPC3 vs VAC3: *P* < 0.001, U = 125376). (**D**) Bar plot with SD of the density of the number of elongated protofilaments and toroidal oligomers per μm^2^ in each sample. The graph shows the significant presence of elongated aggregates in the HPC (n1=35, n2=17, n3=16 per 2020 μm^2^) compared to the VAC samples (n1 = 2, n2 = 0, n3 = 1 per 2020 μm^2^) (Unpaired two-tailed *t*-test, HPC1 vs VAC1: *P* = 0.03, *t*_35_ = 2.195; HPC3 vs VAC3: *P* = 0.02, *t*_15_ = 2.706). (**E**) Statistical analysis of the cross-sectional length of the toroidal, prefibrillar and fibrillar aggregates. (**F**) Statistical analysis of the cross-sectional height of the toroidal, prefibrillar and fibrillar aggregates.

The 3D morphology and the heterogeneity of the aggregates from the HPC and VAC samples were characterized using high-resolution and phase-controlled AFM imaging (Fig. 6A and B).^53–55^ In both the HPC and the VAC samples, we observed the abundant presence of spherical aggregates. The single molecule statistical analysis of the cross-sectional diameter of these aggregates showed that the spherical aggregates present in the HPC samples had a diameter (∼30–50 nm) that was significantly smaller than the diameter of the spherical aggregates in the VAC samples (∼50–80 nm) (Fig. 6C). The difference of the average diameter of the aggregates is in agreement with the results obtained by Aptamer DNA-PAINT in Fig. 5.

Furthermore, we observed that HPC samples contained several elongated toroidal structures, as well as fibrillar and prefibrillar aggregates. The VAC samples contained a significantly smaller number of elongated aggregates, and toroidal aggregates were not found in the 500 *μ*m2 area of the sample that was imaged in a randomized manner. The statistically significant difference in the number of elongated aggregates in the HPC versus VAC samples was evaluated by calculating the density of the number of these elongated aggregates per *μ*m2 (Fig. 6D). The toroidal and fibrillar aggregates had an average length of ∼100 nm (Fig. 6E).

AFM cannot determine the proteins the toroidal, fibrillar and spherical structures consist of. However, the different heterogeneity of the aggregated species in the HPC and VAC samples suggests that there is a regional variability in the structures of soluble aggregates, and toroidal and fibrillar structures might be more toxic than spherical structures.

### Characterization of Aβ-containing fragments through single-molecule pull-down imaging

**Figure 7:**
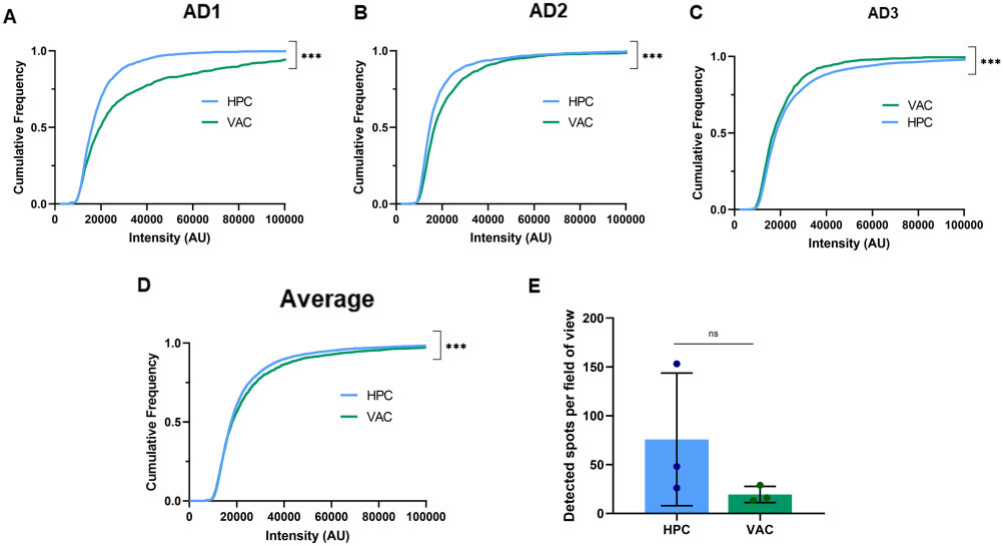
**Single-molecule pull-down characterization of Aβ-containing fragments in samples.** Cumulative frequency plots of intensity values of Aβ aggregates in HPC and VAC samples from (**A**) AD1 (Kolmogorov–Smirnov test, HPC vs VAC, *P* < 0.001, D = 0.1987), (**B**) AD2 (Kolmogorov–Smirnov test, HPC vs VAC, *P* < 0.001, D = 0.1451), (**C**) AD3 (Kolmogorov–Smirnov test, HPC vs VAC, *P* < 0.001, D = 0.0804) and (**D**) Average of the three patients (Kolmogorov–Smirnov test, HPC vs VAC, *P* < 0.001, D = 0.05452). See Supplementary Table 2 for results of multiple comparison test. (**E**) The number of detected spots (Aβ) per field of view (380 x 380 pixel cropped images). Each point represents one of three Alzheimer’s disease patients. Error bars are mean ± SD (Unpaired two-tailed *t*-test, HPC vs VAC, *n* = 3, *P* = 0.47, *t*_4_ = 0.7978).

Aptamer DNA-PAINT and AFM imaging allowed us to characterize the morphology, size, number and shape of the soluble aggregates. However, as the techniques are not protein-specific, the SiMPull technique was employed to specifically characterize the Aβ-containing aggregates in these samples.^56^ Intensity values provide an approximation of aggregate molecular weight, assuming that more fluorescent antibodies bind larger aggregates, and hence an increase in intensity correlates with an increase in aggregate size. The intensity values suggest that VAC samples contain larger Aβ-containing aggregates than the HPC samples in two out of the three patients (Fig. 7A–C), and when averaging the three patients (Fig. 7D). This is in agreement with the Aptamer DNA-PAINT and AFM data, which also show that VAC samples have larger aggregates than HPC samples. There was no significant difference in the number of detectable spots per field of view for the two regions (Fig. 7E).

## Discussion

The only previous study using soluble aggregates extracted from soaking brain tissue analysed Braak stage VI Alzheimer’s disease brains and showed that the major species was Aβ that caused long-term potentiation deficit and neuronal retraction.^23^ In this pilot study, we wanted to first see whether we could detect any aggregates in early disease soluble aggregates, since this would allow us to study earlier events in disease development. We characterized the soluble aggregates from eight different brain regions from three Alzheimer’s disease patients at Braak stage III. Soluble aggregates from all eight regions were neuroinflammatory and liposome-permeable, to varying degrees. This suggests that there is global pathology occurring even at Braak stage III, which is an early stage of disease. We found extensive variation between the same regions in different brains, for example, in the aggregate length and number, but clear differences between HPC and VAC, since the former is affected very early in Alzheimer’s disease and the latter is largely unaffected until late disease. We therefore chose to compare the soluble aggregates in HPC and VAC regions using all assays, with VAC serving as an internal control for each patient.

Despite patient-to-patient variability, HPC aggregates appeared to be the most toxic. TNF-α secretion in response to soluble Aβ aggregates has been previously reported to cause long-term potentiation deficit,^57^ a cellular correlate of memory loss, so together with our data showing inflammation being highest in the HPC, this offers an explanation as to why memory loss occurs in Alzheimer’s disease.

We have identified the size, length, morphology and number of these endogenous soluble aggregates. These varied from region to region and from patient to patient but importantly there was a range of aggregates of different sizes (20–200 nm) in all regions. It should be noted that the aptamer used for the Aptamer DNA-PAINT studies can bind both Aβ and α-synuclein, however, our work^7^^,^^40^ and previous work^23^ suggests that it is most likely Aβ. Furthermore, AFM imaging is not protein specific, but the sizes of the imaged aggregates are consistent with those measured with Aptamer DNA-PAINT, similar to what was observed in our previous work on CSF.^40^

We have taken advantage of the high-resolution of AFM to further characterize the morphology and structure of soluble aggregates from the HPC and the VAC. HPC samples contained structures of toroidal nature, as well as fibrillar structures. VAC samples rarely had fibrillar structures and contained many spherical structures. We have found in CSF^41^ and with synthetic aggregates^7^ that protofibrils are the main inflammatory species. This is because they are the right diameter to be bound by multiple toll-like receptor 4s (TLR4s). Indeed, in the HPC samples about 7–15% of the aggregates detected by AFM have the right height (∼2 nm) to produce a strong inflammatory response, compared to the proportion in the VAC, which is less than 2%. These aggregates were less than 100 nm in length, which is also consistent with our observation, combining our Aptamer DNA-PAINT and inflammatory assay results, that the inflammatory aggregates are less than 100 nm in length. Overall, our data suggest that fibrillary aggregates less than 100 nm in length and 2 nm in diameter can cause inflammation and that there are more of these aggregates in the HPC compared to the VAC.

A previous study has found that activated microglia are present in HPC and VAC in Alzheimer’s disease patients classed as having low neuropathologic change.^10^ Both the HPC and VAC already had some activated microglia. It was found that there are more microglia and more activated microglia in HPC than VAC, but an increase in the proportion of activated microglia occurred in both areas at early stages. This is supported by cross-sectional studies, which have used PET scans to detect activated microglia, and have shown increases in inflammation in early disease all over the brain that is associated with cognitive decline.^58–60^ Peripheral cytokine studies have shown that this increase in inflammation in early disease plateaus in later stages of the disease.^61–64^ Furthermore, recent studies have shown that microglia do not appear to take up and transport soluble Aβ, but instead degrade them by secreting insulin-degrading enzyme (IDE).^65^ In combination with our work, this suggests that instead of aggregates spreading through the brain, the same aggregate-induced inflammation is occurring locally to a greater or lesser extent in the entire brain simultaneously.

It should be noted that our study was intended to determine the feasibility of this approach and has been performed on a small number of Alzheimer’s disease patients due to the manual nature of these experiments. We are working on automating these assays to allow for more high-throughput assessment of Alzheimer’s disease brain tissue, to explore whether similar characteristics of soluble aggregates are found in larger cohorts and allow comparison to age matched control brain and brain at later stages of AD. More sensitive neuroinflammation assays are also needed to better characterize the inflammatory properties of aggregates from different brain regions. In the future, this approach has the potential to characterize the aggregates that form in humans during the development of AD and identify which aggregates are toxic and by what mechanisms. In particular, by studying regions where inflammatory aggregates are just starting to be formed, it may be possible to study the early processes of disease.

Overall, our data are consistent with small soluble Aβ-containing aggregates, 2 nm in diameter and less than 100 nm in length, driving inflammation in Alzheimer’s disease to greater or lesser extents in all regions of the brain and this aggregate-induced inflammation then causing cellular dysfunction and ultimately cell death. Our study also highlights the heterogeneity in size, morphology and structure of the aggregates formed in the brain with the proportion of different aggregates differing between brain regions. It also highlights the challenges in selectively targeting the correct species and suggests that targeting the aggregate induced inflammation may be a better therapeutic strategy than attempting to target specific aggregates.

## Supplementary material

Supplementary material is available at *Brain Communications* online.

## Supplementary Material

fcab147_Supplementary_Data

## Abbreviations

Aβ =: amyloid beta
AFM =: atomic force microscopy
HPC =: hippocampus
PEG =: polyethylene glycol
PVC =: primary visual cortex
SiMPull =: single-molecule pull-down
VAC =: visual association cortex
