# Supplementary material for: Soluble amyloid beta-containing aggregates are present throughout the brain at early stages of Alzheimer’s disease
Source: Brain Commun. 2021 Jul 2;3(3):fcab147. doi: 10.1093/braincomms/fcab147 (PMC8361392; doi:10.1093/braincomms/fcab147)
Supplement: fcab147_Supplementary_Data [file fcab147_supplementary_data.zip › Supplementary_material.pdf]

Supplementary Information

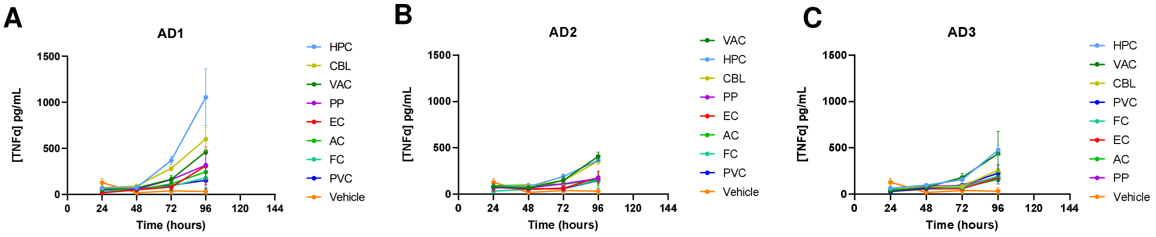

Supplementary Figure 1: Neuroinflammation individual patient data

TNFα response from BV2 cells treated with soluble aggregate samples (diluted 1:5) from eight different brain regions, from three Alzheimer's disease patients: (**A**) AD1, (**B**) AD2, (**C**) AD3. Vehicle control was aCSF at equal volume to soaked brain samples. LPS at 10 ng/mL was used as positive control (not shown). Connecting lines have been added for visual clarity. Error bars are mean ± SD from three wells.

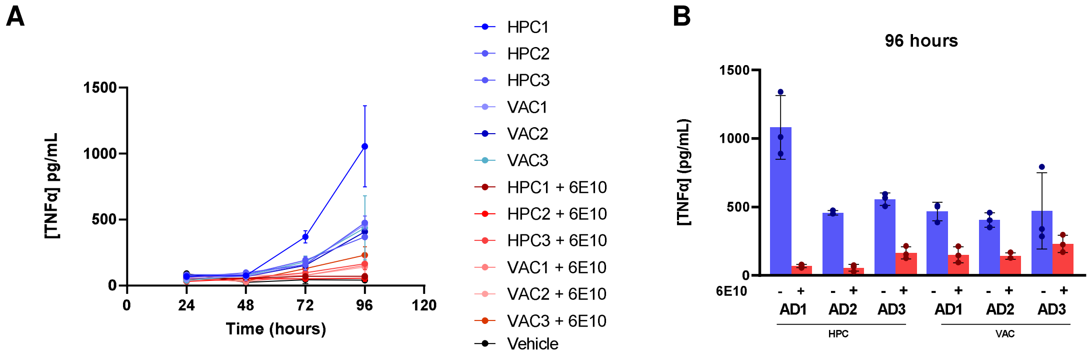

Supplementary Figure 2: Immunodepletion of Aβ-containing fragments individual patient data

(**A**) TNFα response measured from BV2 cells treated with soaked brain samples that have either undergone (red) or not undergone (blue) a pull-down using a 6E10 APP antibody. Vehicle control was aCSF at equal volume to soaked brain samples. LPS at 10 ng/mL was used as positive control (not shown). Error bars are mean ± SD from three wells. The numbers after the regions correspond to the patient (1 = AD1, 2 = AD2, 3 = AD3). (**B**) TNFα measured at the 96 hour timepoint. Error bars are mean ± SD from three wells.

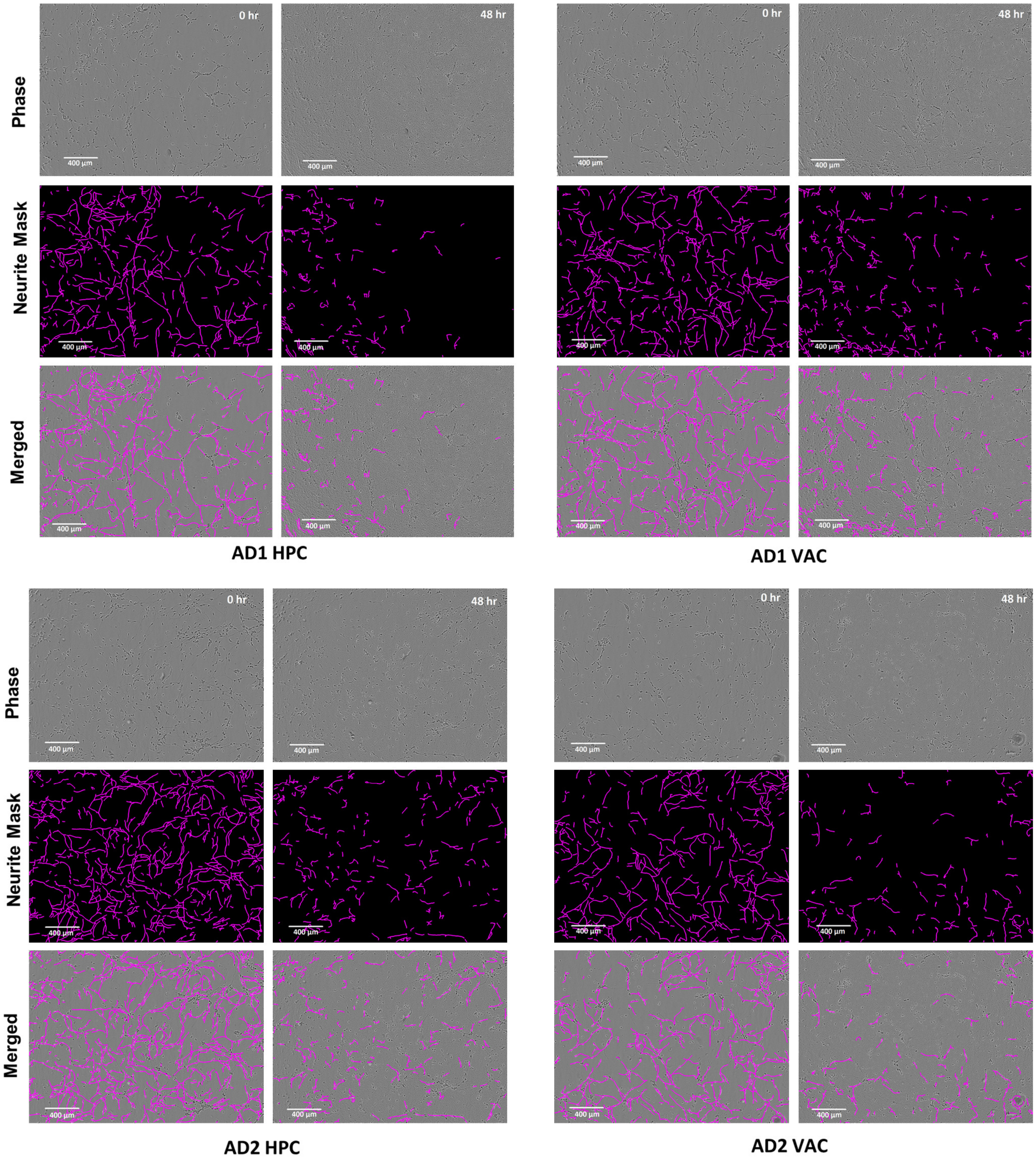

Phase
Neurite Mask
Merged
0 hr
48 hr
400 µm
AD1 HPC
AD1 VAC
AD2 HPC
AD2 VAC

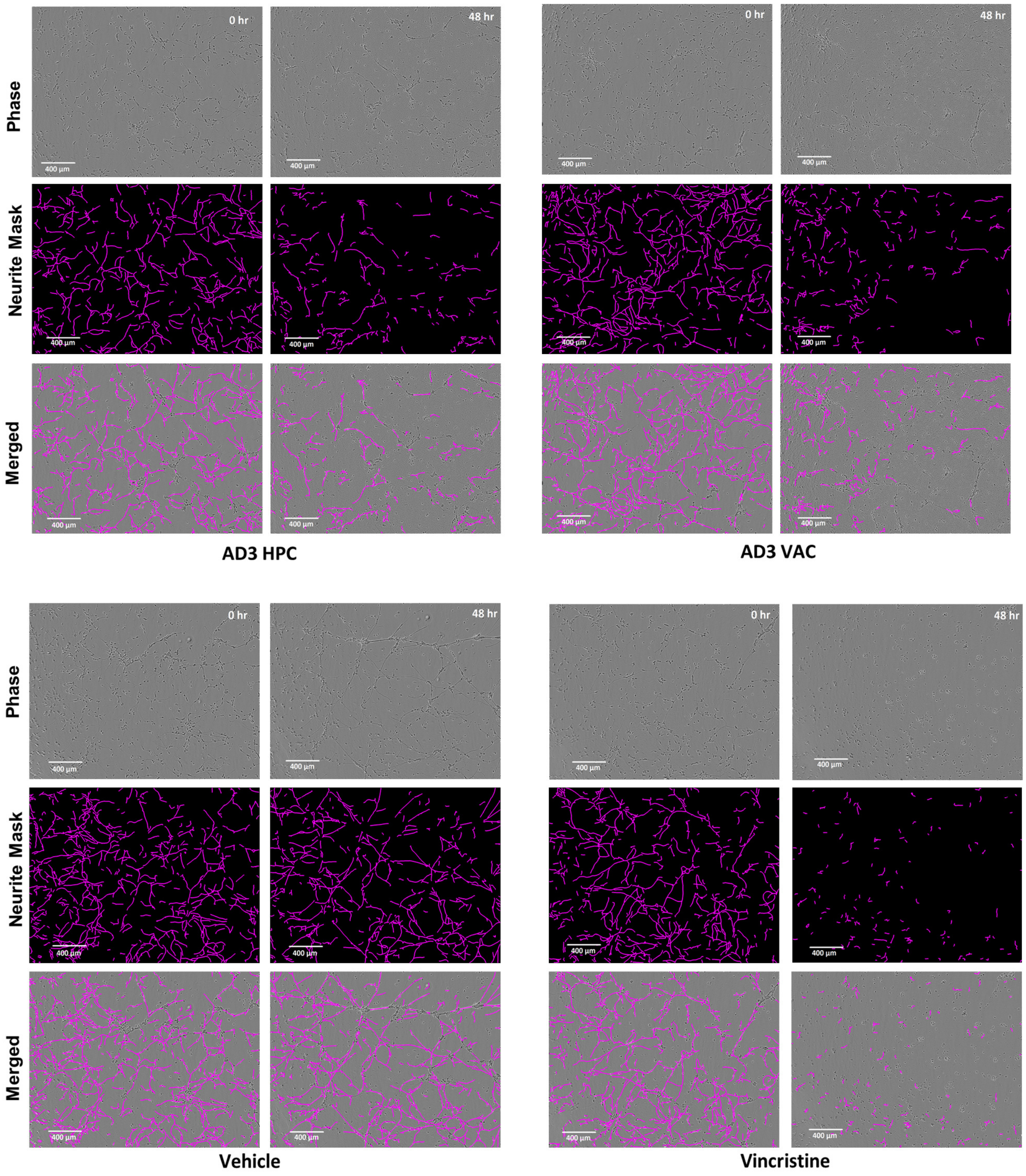

Supplementary Figure 3: Neurite length representative images

Representative images of LUHMES neurons after 48 hours of treatment with aCSF, HPC samples, VAC samples, or Vincristine.

Patient	HPC > VAC	HPC < VAC	Not Significant	No. of tests (#HPC x #VAC)
**AD1**	0	9	0	9 (3 x 3)
**AD2**	0	3	6	9 (3 x 3)
**AD3**	0	9	0	9 (3 x 3)
**Average**	0	9	0	9 (3 x 3)

Supplementary Table 1: Results from multiple comparisons test for Aptamer-DNA PAINT data

HPC > VAC and HPC < VAC refer to cases where the soluble aggregates in the HPC samples were larger or smaller than the VAC aggregates respectively, according to the Kolmogorov-Smirnov test (significance level = 0.01).

Patient	HPC > VAC	HPC < VAC	Not Significant	No. of tests (#HPC x #VAC)
**AD1**	1	1	2	4 (2 x 2)
**AD2**	1	2	1	4 (2 x 2)
**AD3**	3	1	0	4 (2 x 2)
**Average**	2	7	0	9 (3 x 3)

Supplementary Table 2: Results from multiple comparisons test for SiMPull data

HPC > VAC and HPC < VAC refer to cases where the soluble aggregates in the HPC samples were larger or smaller than the VAC aggregates respectively, according to the Kolmogorov-Smirnov test (significance level = 0.01).
