# Supplementary material for: Soluble amyloid beta-containing aggregates are present throughout the brain at early stages of Alzheimer’s disease
Source: Brain Commun. 2021 Jul 2;3(3):fcab147. doi: 10.1093/braincomms/fcab147 (PMC8361392; doi:10.1093/braincomms/fcab147)
Supplement: fcab147_Supplementary_Data [file Original Submission - Authors Response.pdf › Original Submission - Authors Response.pdf]

Reviewer(s)' Comments to Author:

Reviewer: 1

Comments to the Author

The paper is well written and illustrated but has one crushing flaw for this reviewer: the control used throughout is only the vehicle (aCSF) and there is no "normal" brain control for the toxicity experiments. This is needed if they wish to establish that the toxic molecules are indeed their Abeta containing ones related to the disease progression.

We thank you for your comments. We agree and are aware of this limitation, however it should be noted that the process of extracting the soluble aggregates and carrying out our assays is very time and resource intensive. The data for this paper has been generated by a large collaborative effort spanning the last 2-3 years. More importantly, finding a suitable negative control (or “normal” brain) is not trivial, as it requires finding an age-matched control who has not passed away from brain/spinal cord injuries/illnesses, and has no AD pathology or cognitive impairment. This is complicated further by the fact that non-demented individuals, especially at the age of 70 and above, often present substantial plaques and tangles without cognitive issues. It is also important to note that the AD brains analysed in this study were from Braak stage III (early disease) and are therefore unlikely to display significant differences to age-matched non-demented brains that will also have pathology. Studies focusing on later stages of disease (e.g. Braak stage VI) would benefit more from a comparison with age-matched control brain, as the differences in pathology would be significant. Due to the complexity of finding suitable negative control brain, we decided it would be more relevant to use a region affected in the later stages of the disease (visual association cortex) as an internal control for each brain.

Regarding the relationship of Aβ-containing molecules and disease progression, we have not measured how aggregates change during disease in this study. This could only be confirmed by characterising the soluble aggregates in more Braak stages and would be the subject of a future study.

I am also troubled by their lumping the three brains together considering that the patient’s cause of death was cancer, pneumonia and only the 3rd, the oldest seems to have been directly related to AD. Further, I would have expected some difference in the VAC results just based on age.

We agree, which is why individual patient data has also been presented for each figure and has shown similar trends to the averaged data. Furthermore, the death of AD patients is often due to illness-related complications such as infections or blood clots, and not always directly due to AD itself. In fact, the death certificate will often state AD when the proximate cause of death is an infective complication such as pneumonia. It is therefore difficult to obtain AD brains, from similar stages of pathology (Braak Stage), all with the same cause of death. The brains we have used are all from patients confirmed by a neuropathologist to have been at Braak Stage III at time of death. Regarding the age-related changes in VAC, as far as we’re aware these experiments have not been done before so we had no expectations going in.

Other than that, I find the level of experimental detail very good and the methods clearly defined. I especially like Fig. 1, which explains in a page all the experiments they have done to correlate their data. There are a few minor details (i.e., on p. 7, line 1 they should mention the patient details table 1 (which in a rewrite should also include at least one age matched negative control patient).; p.12, line 42, they should note the pbs will contain the sample). I was also puzzled on p.7 why the air around them would be LPS contaminated and why this would be given a reason for protecting their pipettes with filtered tips.

We have made the following additions/changes: we have referenced the patient information table on p.7, line 1; we have changed ‘under a fume hood’ to ‘sterile conditions’, and ‘filtered’ to ‘pre-sterilised’ on p.7.

Regarding p.12, line 42, the PBS does not contain the soluble aggregate samples; the samples are added earlier in the protocol. At this final step before imaging, only PBS is added (without sample), to create a seal between the two coverslips. To make this clearer, the sentence has been changed to “to properly seal the imaging chamber and prevent evaporation, 3 µL of PBS was added to each well and sandwiched with a second coverslip.”

Reviewer: 2

Comments to the Author

The manuscript by Sideris et al presents a very advanced technical platform to study the distribution, size and effects of Aβ-containing aggregates. However, a number of limitations raise concerns on the conclusions as presented.

Some statements such as "Aβ is driving the soaked brain inflammatory response" are too strong. In this particular case, a main concern is the use of 6E10 for the immunoprecipitation. 6E10 is not a Aβ-specific antibody but will pulldown any APP-derived fragment containing the Aβ portion. Therefore, the authors cannot ascertain the effects of Aβ in relation to other Aβ-containing fragments (full-length APP, C99, etc) see paper by Hunter and Brayne 2017 (J Negat Results Biomed;16:1). The authors should clarify their choice over an Aβ-specific antibody, which does not cross-react with the full-length APP precursor.

We agree, the 6E10 antibody cannot distinguish between Aβ and APP. To address this, we have changed our terminology, from Aβ to Aβ-containing fragments, where appropriate. However, it should be noted that APP is supposed to be membrane bound, and we are looking at the soluble fraction using minimally perturbative methods. Furthermore, the species of Aβ in AD brain are very heterogenous. To avoid discriminating against certain species of Aβ, we used the 6E10 APP binding antibody, which is very widely used and binds to all Aβ-containing fragments. We considered using other widely used Aβ-specific antibodies such as 4G8, but as stated in Hunter and Brayne 2017 (J Negat Results Biomed;16:1), these antibodies often cross react with other proteins including α-synuclein.

Additionally, some methodological steps need clarification. The terminology is quite confusing. Are "soaked brain aggregates" the same thing as "soluble aggregates" as figure 1 suggests? More importantly, the extraction of soluble aggregates from human brain tissue involves a 72-hour dialysis step. As Aβ aggregates spontaneously, how do the authors ensure that the soluble aggregates gathered at the end of the purification do reflect the species present in the brain? This might be a confounding for the results presented in figures 5 and 6.

We have changed “soaked brain aggregates” to “soluble aggregates” throughout the paper to keep the terminology consistent. The levels of monomer and soluble aggregates in these samples are extremely low, picomolar or below (they could not be determined using western blot or ELISA, only by the more sensitive SiMPull method), and the samples were dialysed in 4 °C. Therefore, due to the low monomer concentration and low temperature no further aggregation can occur during this step. The dialysis step is necessary for the removal of molecules under 2.5 kDa such as damage-associated molecular patterns (DAMPs), which could affect inflammation and cytotoxicity experiments. However, it would be interesting if future optimisations of the brain soaking method could aim to shorten the length of the dialysis step.

It should also be noted that aggregates are stable once formed, and we are only looking at aggregates stable for 72h.

Also of importance is the fact that the cause of death of patient AD3 is end-stage dementia. This suggests that in addition to tau staging, more information is needed on the neuropathology of these individuals and to determine if such information correlates with the data presented, especially since the authors do acknowledge regarding figure 5 that "The aggregates vary from region to region, and from patient to patient". It is clear that AD3 has more extensive pathology explaining the smaller differences between HPC and VAC regions (Fig 5B).

This is a good point, and we agree that more information about neuropathology would be useful, however since our sample size is so small, this may not be the only reason why there are smaller differences in HPC and VAC for patient AD3. A much more complete study is required to be able to explain this result.

Lastly, the manuscript is missing some important recent literature on inflammation and soluble Aβ.

Thank you. It is not clear exactly what references the reviewer has in mind but we have added some additional recent citations to the paper. These are:

Kinney JW, Bemiller SM, Murtishaw AS, Leisgang AM, Salazar AM, Lamb BT. Inflammation as a central mechanism in Alzheimer’s disease. *Alzheimer’s Dement Transl Res Clin Interv*. 2018;4:575-590.

Li S, Selkoe DJ. A mechanistic hypothesis for the impairment of synaptic plasticity by soluble Aβ oligomers from Alzheimer’s brain. *J Neurochem*. 2020;154:583-597.

Fu H, Liu B, Li L, Lemere CA. Microglia Do Not Take Up Soluble Amyloid-beta Peptides, But Partially Degrade Them by Secreting Insulin-degrading Enzyme. *Neuroscience*. 2020;443:30-43.

Hector A, Brouillette J. Hyperactivity Induced by Soluble Amyloid-β Oligomers in the Early Stages of Alzheimer's Disease. *Front Mol Neurosci*. 2021;13:1-15.

Corbett GT, Wang Z, Hong W, et al. PrP is a central player in toxicity mediated by soluble aggregates of neurodegeneration-causing proteins. *Acta Neuropathol*. 2020;139:503-526.

Editorial Office comments:

-Please supply a graphical abstract. A graphical abstract is a visual representation of the central finding or methodology of the paper. It will be displayed in the table of contents. Authors should strive to make the graphical abstract informative, interesting, visually appealing, and straightforward (no caption needed, no abbreviations except gene names). Please upload as a separate high resolution image file (tiff preferred). The technical requirements for the graphical abstracts are: --size of the submitted image: 1200 pixels square at 300dpi --font of the text: Arial 12-16 point (smaller fonts will not be legible online)

A graphical abstract has been provided.

-Please add an Abbreviated Summary (up to 50 words) of your paper that captures the main purpose and conclusions of the work. The summary should state the main results written in the third person ('[1st author surname] et al. report that ...') and conclusion. This summary will be used in the contents list online and should not contain any abbreviations other than those on the accepted abbreviations list and accepted gene/protein names, making it accessible to the non-specialist. Please upload it as a Word document. Allowed abbreviations: AIDS; ANOVA; AMPA; ATP; A,T,C,G; CNS; CSF; CT; DNA; ECG; EEG; EMG; GABA; HIV; MRI; NMDA; PET; PCR; RNA.

Sideris et al. report that soluble inflammatory amyloid beta-containing aggregates are found in all regions of early Alzheimer's disease brain. Aggregation may therefore be occurring all over the brain simultaneously, with different brain regions being in different stages of the same process, with the later stages causing increased inflammation.

- To comply with our ethical standards, the appropriate checklist must be included (see Reporting Guideline section within 'Instructions to authors').

As this is a case-control study, we have attached the STROBE checklist.

- When comparing data with more than 2 groups, please use ANOVA or other appropriate test (not-t-test).

We have made the appropriate changes regarding the statistical analysis (Fig. 2C, 4D).
