# Supplementary material for: Soluble amyloid beta-containing aggregates are present throughout the brain at early stages of Alzheimer’s disease
Source: Brain Commun. 2021 Jul 2;3(3):fcab147. doi: 10.1093/braincomms/fcab147 (PMC8361392; doi:10.1093/braincomms/fcab147)
Supplement: fcab147_Supplementary_Data [file Original Submission - Decision Letter.pdf › Original Submission - Decision Letter.pdf]

Original submission – decision letter

19-Apr-2021

Dear Dr. Klenerman,

Thank you for your recent submission BRAINCOM-2021-105 entitled "Soluble inflammatory Aβ aggregates are present throughout the brain at early stages of Alzheimer's disease" to Brain Communications.

Your manuscript has been reviewed, and while the reviewers and editorial team appreciate many aspects of the paper, they raise some concerns that you will need to address in order for the paper to be considered for publication.

If you can address these concerns, I invite you to submit a revised manuscript and a response to each of the reviewers' comments. The reviewer(s)' comments are included at the bottom of this message.

In addition to the reviewers' comments, please note our editorial comments added below to comply with our requirements for presentation of scientific data at Brain Communications.

Please format your revised manuscript according to our Instructions to Authors https://academic.oup.com/braincomms/pages/General_Instructions – a summary of key points is attached to help with this.

To revise your manuscript, click the link below or log into https://mc.manuscriptcentral.com/braincom and enter your Author Centre, where you will find your manuscript title listed under "Manuscripts with Decisions." Under "Actions," click on "Create a Revision." Your manuscript number will be appended to denote a revision.

*** PLEASE NOTE: This is a two-step process. After clicking on the link, you will be directed to a webpage to confirm. ***

https://mc.manuscriptcentral.com/braincom?URL_MASK=063ba63ae38c4ac4a941896393a0e7dc

Please upload a revised manuscript with changes tracked with track changes or in coloured text.

When submitting your revised manuscript, you will be asked to respond to the reviewer(s) comments. Please be as specific as possible in your response to all of the comments.

The editorial team at Brain Communications aims to increase transparency in scientific publishing and to demonstrate the value that peer review brings to the scientific process. Please therefore consider whether you are willing to have reviewer(s)' comments on your paper and your response published as supplementary data with the paper if it is accepted. This is entirely optional and will only be done if both the authors and reviewers agree. While submitting your revision there will be an opportunity to review or update the answer you gave previously, if necessary.

IMPORTANT: Your original files are available to you when you upload your revised manuscript. Please delete any redundant files before completing the submission.

Because we are trying to facilitate timely publication of manuscripts submitted to Brain Communications, we would appreciate your revised manuscript as soon as possible. If it is not possible for you to resubmit within 3 months, the option to submit a revision will expire, and we would then consider your revised paper as a new submission. However, if you need a little more time, please get in touch and we can extend this deadline for you.

Once again, thank you for submitting your manuscript to Brain Communications and I look forward to receiving your revision.

Yours sincerely,

Dr. Rakez Kayed
Editor, Brain Communications

Reviewer(s)' Comments to Author:

Reviewer: 1 (Schien, Catherine)

Comments to the Author
The paper is well written and illustrated but has one crushing flaw for this reviewer: the control used throughout is only the vehicle (aCSF) and there is no "normal" brain control for the toxicity experiments. This is needed if they wish to establish that the toxic molecules are indeed their Abeta containing ones related to the disease progression. I am also troubled by their lumping the three brains together considering that the patients cause of death was cancer, pneumonia and only the 3rd, the oldest seems to have been directly related to AD. Further, I would have expected some difference in the VAC results just based on age.
Other than that, I find the level of experimental detail very good and the methods clearly defined. I especially like Fig. 1, which explains in a page all the experiments they have done to correlate their data.
There are a few minor details (i.e., on p. 7, line 1 they should mention the patient details table 1 (which in a rewrite should also include at least one age matched negative control patient).; p.12, line 42, they should note the pbs will contain the sample). I was also puzzled on p.7 why the air around them would be LPS contaminated and why this would be given a reason for protecting their pipettes with filtered tips.

Reviewer: 2

Comments to the Author
The manuscript by Sideris et al presents a very advanced technical platform to study the distribution, size and effects of Aβ-containing aggregates. However, a number of limitations raise concerns on the conclusions as presented.

Some statements such as “Aβ is driving the soaked brain inflammatory response” are too strong. In this particular case, a main concern is the use of 6E10 for the immunoprecipitation. 6E10 is not a Aβ-specific antibody but will pulldown any APP-derived fragment containing the Aβ portion. Therefore, the authors cannot ascertain the effects of Aβ in relation to other Aβ-containing fragments (full-length APP, C99, etc) see paper by Hunter and Brayne 2017 (J Negat Results Biomed;16:1). The authors should clarify their choice over an Aβ-specific antibody, which does not cross-react with the full-length APP precursor.

Additionally, some methodological steps need clarification. The terminology is quite confusing. Are “soaked brain aggregates” the same thing as “soluble aggregates” as figure 1 suggests? More importantly, the extraction of soluble aggregates from human brain tissue involves a 72-hour dialysis step. As Aβ aggregates spontaneously, how do the authors ensure that the soluble aggregates gathered at the end of the purification do reflect the species present in the brain? This might be a confounding for the results presented in figures 5 and 6.

Also of importance is the fact that the cause of death of patient AD3 is end-stage dementia. This suggests that in addition to tau staging, more information is needed on the neuropathology of these individuals and to determine if such information correlates with the data presented, especially since the authors do acknowledge regarding figure 5 that “The aggregates vary from region to region, and from patient to patient”. It is clear that AD3 has more extensive pathology explaining the smaller differences between HPC and VAC regions (Fig 5B).

Lastly, the manuscript is missing some important recent literature on inflammation and soluble Aβ.

Editorial Office comments:

-Please supply a graphical abstract. A graphical abstract is a visual representation of the central finding or methodology of the paper. It will be displayed in the table of contents. Authors should strive to make the graphical abstract informative, interesting, visually appealing, and straightforward (no caption needed, no abbreviations except gene names). Please upload as a separate high resolution image file (tiff preferred). The technical requirements for the graphical abstracts are:
--size of the submitted image: 1200 pixels square at 300dpi
--font of the text: Arial 12-16 point (smaller fonts will not be legible online)

-Please add an Abbreviated Summary (up to 50 words) of your paper that captures the main purpose and conclusions of the work. The summary should state the main results written in the third person ('[1st author surname] et al. report that …') and conclusion. This summary will be used in the contents list online and should not contain any abbreviations other than those on the accepted abbreviations list and accepted gene/protein names, making it accessible to the non-specialist. Please upload it as a Word document. Allowed abbreviations: AIDS; ANOVA; AMPA; ATP; A,T,C,G; CNS; CSF; CT; DNA; ECG; EEG; EMG; GABA; HIV; MRI; NMDA; PET; PCR; RNA.

- To comply with our ethical standards, the appropriate checklist must be included (see Reporting Guideline section within 'Instructions to authors').

- When comparing data with more than 2 groups, please use ANOVA or other appropriate test (not-t-test).
