# Supplementary material for: Soluble amyloid beta-containing aggregates are present throughout the brain at early stages of Alzheimer’s disease
Source: Brain Commun. 2021 Jul 2;3(3):fcab147. doi: 10.1093/braincomms/fcab147 (PMC8361392; doi:10.1093/braincomms/fcab147)
Supplement: fcab147_Supplementary_Data [file Revision 1- Decision Letter.pdf › Revision 1- Decision Letter.pdf]

Revision 1 – decision letter

17-May-2021

Dear Dr. Klenerman,

It is a pleasure to accept your manuscript entitled "Soluble amyloid beta-containing aggregates are present throughout the brain at early stages of Alzheimer's disease" for publication in Brain Communications.

The next step is for your paper to go through "First Look". We will contact you again shortly if any final files or formatting changes are required for publication or if we have any queries.

If no changes are needed, your submission will be transferred to our Production Department and you will hear from the Production Editor in due course.

Video abstracts - Brain Communications encourages authors to provide a video abstract to accompany their article. These are hosted on the Brain/Brain Communications YouTube channel. If you are interested in producing a 5-min video abstract to promote your article, then please contact the Editorial Office (brcoms.editorialoffice@oup.com) for further details and guidelines.

Thank you very much for your contribution. On behalf of the Editors of Brain Communications, we look forward to your future contributions to the Journal.

Yours sincerely,

Dr Rakez Kayed
Associate Editor
Brain Communications

Professor Tara Spires-Jones
Founding Editor, Brain Communications
